# Calcium-dependent protein kinase CDPK16 phosphorylates serine-856 of glutamate receptor-like GLR3.6 protein leading to salt-responsive root growth in Arabidopsis^#^

**DOI:** 10.3389/fpls.2023.1093472

**Published:** 2023-02-03

**Authors:** Dhanasekar Silamparasan, Ing-Feng Chang, Tsung-Luo Jinn

**Affiliations:** ^1^ Institute of Plant Biology, National Taiwan University, Taipei, Taiwan; ^2^ Department of Life Science, National Taiwan University, Taipei, Taiwan

**Keywords:** 14-3-3Ω, CDPK16, glutamate receptor-like protein 3.6, salt stress, root growth

## Abstract

Calcium-permeable channels in the plasma membrane play vital roles in plant growth, development, and response to environmental stimuli. Arabidopsis possesses 20 glutamate receptor-like proteins that share similarities with animal ionotropic glutamate receptors and mediate Ca^2+^ influx in plants. Calcium-dependent protein kinases (CDPKs) phosphorylate serine (Ser)-860 of glutamate receptor-like (GLR)3.7 protein, which interacts with 14-3-3ω and plays an essential role in salt and abscisic acid response in Arabidopsis by modulating Ca^2+^ signaling. However, the significance of CDPK- mediated phosphorylation status of Ser residues of GLR3.6 with regard to the functioning of GLR3.6 remains to be elucidated. In this study, we performed an *in vitro* kinase assay using CDPK16 and peptides containing the 14-3-3ω interacting domain of GLR3.6. We showed that Ser861/862 of GLR3.6 are required for the interaction with 14-3-3ω and that Ser856 of GLR3.6 is specifically phosphorylated by CDPK16 but not by CDPK3 and CDPK34. In addition, the expression of *GLR3.6* was quickly downregulated by salt stress, and plants of *glr3.6* mutants and *GLR3.6*-overexpression lines presented shorter and longer root lengths, respectively, under normal growth conditions than Col. Overexpression of the GLR3.6-Ser856 to Ala mutation resulted in a less sensitive phenotype in response to salt stress similar to *glr3.6*. Our results indicated that the Ser861/862 residues of GLR3.6 are required for interaction with 14-3-3ω. Additionally, the phosphorylation status of Ser856 residue of GLR3.6, which is mediated specifically by CDPK16, regulates root growth in normal and salt stress and conditions.

## 1 Introduction

Glutamate acts as an excitatory neurotransmitter in the central nervous system of animals and facilitates long-range information exchange *via* the activation of ionotropic glutamate receptors (iGluRs) (Lam et al., 1998; Grenzi et al., 2022). iGluRs are ligand-gated non-selective cation channels that influx Ca^2+^, Na^+^, and K^+^ during neuronal signaling in addition to playing an essential role in psychosis, Alzheimer’s disease, and neurological disorders (Vignes and Collingridge, 1997; Stawski et al., 2010; Flores-Soto et al., 2012; Hansen et al., 2021). Glutamate receptor-like proteins have also been identified in plants and *Chlamydomonas* (Lam et al., 1998; Chiu et al., 2002; Green et al., 2021) and share a substantial similarity in protein structure to that of iGluRs found in animals (Price et al., 2012; Xue et al., 2022). Genomic analysis indicated that 20 glutamate receptor-like (GLR) coding sequences found in Arabidopsis were divided into three groups, GLR1, GLR2, and GLR3 (Green et al., 2021; Naz et al., 2022). Recent studies have shown that Arabidopsis GLRs are selective cation channels and potential candidates for Ca^2+^ influx in plants (Singh et al., 2016; Cheng et al., 2018; Wang et al., 2019; Xue et al., 2022).

Ca^2+^ is an essential secondary messenger in both animal and plant cells and has been linked to the perception of environmental stress (Tuteja and Sopory, 2008; Meena et al., 2019; Park and Shin, 2022). The homeostasis of cytosolic Ca^2+^ concentration is a factor in diverse cellular pathways and responses to numerous stimuli (Mahajan et al., 2006; Luan and Wang, 2021). The EF-hand motif is the most common Ca^2+^-binding motif found in proteins, particularly in calcium-dependent protein kinases (CDPKs), and the binding of Ca^2+^ to CDPKs correlates with physiological responses (Kretsinger and Nockolds, 1973; Carafoli, 1994; Dekomah et al., 2022).

Various Arabidopsis GLRs perform varied functions, such as: GLR3.1 in stomatal closure (Cho et al., 2009); GLR1.2 and GLR3.7 in pollen tube morphogenesis (Michard et al., 2011); GLR3.4, GLR3.5, and GLR3.7 in abscisic acid (ABA) biosynthesis and salt stress-response during seed germination (Kong et al., 2015; Cheng et al., 2018; Wang et al., 2019; Chen et al., 2021; Green et al., 2021); GLR3.4 in touch and cold stress-signaling (Meyerhoff et al., 2005); GLR3.4 and GLR3.6 in lateral root initiation and development (Vincill et al., 2013; Singh et al., 2016); GLR3.3, GLR3.5, and GLR3.6 in innate immune and aphid feeding responses (Forde and Roberts, 2014; Vincent et al., 2017); and GLR3.2, GLR3.3, GLR3.5, and GLR3.6 in wound-induced signaling (Mousavi et al., 2013; Salvador-Recatala, 2016; Nguyen et al., 2018; Toyota et al., 2018; Xue et al., 2022). Despite their physiological functions, GLRs are detected in diverse subcellular localizations, such as the plasma membrane and membranes of chloroplasts, mitochondria, and vacuoles (Teardo et al., 2011; Wudick et al., 2018; Hansen et al., 2021).

The 14-3-3 protein was initially discovered in mammalian brain tissue and exists in all eukaryotes (Foote and Zhou, 2012). They function as scaffold proteins and occur as homo- or heterodimers. They interact with phosphoproteins to regulate protein functions and affect diverse cellular processes (Chang et al., 2009; Huang et al., 2022). In Arabidopsis, 14-3-3 protein has 13 isoforms (Rosenquist et al., 2001; Sehnke et al., 2002; Paul et al., 2005; Camoni et al., 2018), and their target binding sites include K/RXXS^p^/T^p^XP (Mode 1), K/RXXXS^p^/T^p^XP (Mode 2), and YT^p^V (Mode 3), and other non-consensus motifs have been recently discovered (Huang et al., 2013). The S^p^/T^p^ stands for phosphorylated Ser/Thr, and X represents any amino acid. Arabidopsis GLR1.3, -1.4, -2.6, -2.9, -3.6, and -3.7 are predicted to have a 14-3-3 (κ, ψ, ω, and χ isoform) binding motif and most display a Mode-1 or -2 binding pattern (Chang et al., 2009; Shin et al., 2011; Wudick et al., 2018); therefore, 14-3-3 interactions might be a common feature among most members of the GLR family in plants. However, only GLR3.7 has been experimentally shown to interact with 14-3-3ω (Wudick et al., 2018; Wang et al., 2019). Potential 14-3-3 binding sites could be logical candidates for CDPK substrates because CDPK phosphorylation sites are implicated in many 14-3-3 binding motifs (Chang et al., 2009; Huang et al., 2013; Ito et al., 2014; Chang et al., 2019; Wang et al., 2019). However, the binding and function of these target proteins remain poorly characterized.

In Arabidopsis, 34 CDPKs (divided into four subgroups) and 8 plants-specific CDPK-related kinases (CRKs) have been identified (Harper et al., 1994; Hrabak et al., 2003; Mori et al., 2006; Zhao et al., 2011; Rigó et al., 2013; Li et al., 2018). CDPKs have been implicated in multiple aspects of plant growth, development, and stress response (Klimecka and Muszynska, 2007; Ishida et al., 2008; Geiger et al., 2010; Bredow et al., 2021). Interestingly, subgroup-IV CDPKs such as CDPK16, -18, and -28 are closely connected with CRKs. Recent studies have shown that CRKs positively regulate root growth and gravitropism (Harper et al., 1991; Lewis et al., 2011; Baba et al., 2018; Yip Delormel and Boudsocq, 2019; Dekomah et al., 2022). GLR3.6 controls root development as evidenced by the smaller root length phenotype observed in *GLR3.6*-mutated plants (Singh et al., 2016). Additionally, [Ca^2+^]_cyt_ was reduced in GLR3.6 mutants, suggesting impaired Ca^2+^ signaling (Singh et al., 2016). However, the mechanism underlying the CDPK-mediated phosphorylation of GLR3.6 is yet to be elucidated.

In this study, *in vitro* kinase assay was performed using CDPK16 and GST-fused peptides of the GLR3.6 fragment (amino acids 854-EGSIRRRSSPSA-865) and its variants harboring on the Ser to Ala mutation on the 14-3-3ω interacting domain. We showed that Ser856 of GLR3.6 is specifically phosphorylated by CDPK16 but not by CDPK3 or CDPK34. Additionally, the unknown kinase phosphorylated Ser861/862 residues of GLR3.6 is required for interaction with 14-3-3ω.

## 2 Materials and methods

### 2.1 Plant materials and growth conditions

*Arabidopsis thaliana* Columbia ecotype (Col) plants were used for the experiments. T-DNA insertional mutants of *glr3.6-1* (SALK 091801C) and *glr3.6-2* (SALK 035353) were obtained from the Arabidopsis Biological Resource Center (ABRC, Ohio State University, Columbus, United States). Seeds were sterilized with bleach (3%) and kept in the dark for 3 days at 4°C and then sown on 1/2 Murashige and Skoog (MS) medium plates containing 0.8% agar and 1% sucrose. The sown seeds were grown under long-day photoperiod conditions (16 h light/8 h dark) at 22°C and 80–100 μmol m^-2^ s^-1^ light intensity. *GLR3.6*-overexpression lines were generated by *Agrobacterium tumefaciens* GV3101-mediated transformation with the pEarleyGate101 vector using the floral dip method (Clough and Bent, 1998).

### 2.2 Primary root growth assay

The primary root length was assayed as previously described (Singh et al., 2016). Seedlings were grown vertically on 1/2 MS plates containing 0.8% agar and 1% sucrose for 4 days, transferred to 1/2 MS plates containing 100 or 125 mM NaCl respectively, and grown vertically for 6 days under 16 h light/8 h dark photoperiod condition in a plant-growth chamber. ImageJ software (https://imagej.nih.gov/ij/) was used to measure primary root length.

### 2.3 RNA preparation, cDNA synthesis, and quantitative real-time PCR (q-PCR)

RNA was extracted from 7-day-old seedlings using REzolTM C&T reagent (Protech, Taipei, Taiwan). A TURBO DNA-free kit (Applied Biosystems) was used to remove contaminating DNA. A high-capacity cDNA reverse transcription kit (Applied Biosystems-Thermo Fisher Scientific) was used to synthesize cDNA from 2 µg of the extracted RNA. qPCR was performed using Taq DNA Polymerase 2x Master Mix (Ampliqon) and the KAPA SYBR FAST Universal Kit (Sigma-Aldrich).

### 2.4 Fusion peptide design and construction of glutathione-S-transferase (GST)- and His-tagged proteins

The fusion peptide primers for GLR3.6 and GLR3.7 were designed as previously described (Curran et al., 2011; Wang et al., 2019) to self-anneal around a 45–60 nucleotide sequence harboring the *Asc*I and *Not*I restriction sites conducive for cloning into the GST-NRV vector (**Appendix 1A**). The GST-NRV vector-only control produced proteins containing the GST-RFP-Strep tag II. For generating the 6xHis-14-3-3ω tagged protein, the full-length 14-3-3ω sequence was acquired and cloned into a 6xHis-tag vector pRSETA using the *Bam*H1 and *Eco*R1 restriction sites (**Appendix 1B**), as previously reported by Chen et al. (2018). The CDPKs were cloned into the pGEX4T-1-6xHis vector as previously described by Wang et al. (2019) (**Appendix 1C**) to produce GST-CDPKs-6xHis. The plasmids were transformed into BL21 cells for recombinant protein expression.

### 2.5 Purification of 6xHis- and GST-tagged proteins

IPTG-induced BL21 cells (200 mL) were sonicated, and the cell lysate was centrifuged at 9000 × *g* (Beckman Coulter J2-MC, USA) at 4°C for 30 min. The supernatant was incubated in 500 μL of Ni-NTA agarose (Profinity IMAC NI-charged Resin, 1560131) at 4°C for 2 h. The agarose was washed five times with buffer containing 20 mM Tris (pH 8.0), 10 mM imidazole, 500 mM NaCl, and 10% glycerol, followed by a second wash with buffer containing 20 mM Tris (pH 8.0) and 100 mM NaCl. To elute the 6xHis-tagged proteins, 1 mL elution buffer containing 300 mM imidazole, 100 mM NaCl, and 20 mM Tris (pH 8.0) was used.

For purification of the GST-tagged protein, 500 μL of glutathione resin (Omics Bio, CL00206) was washed with PBS buffer. The resin was then added to the sample with 1% NP-40 and incubated at 4°C for 2 h. The mixture was transferred to bio-spin columns (Bio-Rad), washed five times with PBS buffer, followed by elution with 500 μL of elution buffer containing 20 mM glutathione, 150 mM NaCl, and 175 mM Tris buffer (pH 8.0). The amount of protein was quantified using the Bradford assay (Bradford, 1976).

### 2.6 Site-directed mutagenesis

The QuikChange Lightning kit (Agilent Technologies; 210518) was used to generate Ser to Ala point mutations in GLR3.6, as previously reported (Huang et al., 2013). Two complementary oligonucleotides containing the desired mutation and flanked by an unmodified nucleotide sequence were designed. The mutated nucleotides were amplified using PCR, and the *Dpn*I restriction enzyme was added to the amplification reaction, followed by incubation at 37°C for 5 min to digest the parental double-stranded DNA. *Dpn*I-treated DNA was then transformed into DH5α competent cells.

### 2.7 *In vitro* kinase assay

The *in vitro* kinase assay was performed as previously described (Wang et al., 2019). A reaction mixture (18 μL) containing 0.40 μg of GST-CDPK-6xHis kinase, 5–10 μg of substrates of GST-fused peptides GLR3.6 or GLR3.7, and 2 μL of 10X buffer (200 mM Tris pH 7.5, 100 mM MgCl2, 10 mM EGTA, and 11 mM CaCl_2_) was used. Next, 2 μL of 50 μM ATP solution (spiked with 2.5 μCi [γ-^32^P] ATP) was added to initiate the kinase reaction at room temperature (24–28°C) for 10 min. The reaction was stopped by adding 5 μL of 4X sodium dodecyl sulfate (SDS) sample buffer. Samples were analyzed using 10% SDS-PAGE. The γ-^32^P-labeled signals were normalized to the amount of protein determined using Coomassie brilliant blue-stained gels. The γ-^32^P-labeled signals were detected using an image analyzer (GE Healthcare, Typhoon 9400).

### 2.8 Protoplast isolation, transient expression, and bimolecular fluorescence complementation (BiFC) assay

Arabidopsis protoplasts were isolated from four-week-old plant leaves using a previously described (Yoo et al., 2007). Protoplasts (3 × 10^4^ cells) were transfected with 10–20 μg of plasmid DNA and then incubated at 22°C for 16–24 h. Agroinfiltration-based transient gene expression in tobacco (*Nicotiana benthamiana*) leaves was performed as previously described (Wang et al., 2019). Agrobacteria (GV3101) were infiltrated into 4-week-old tobacco leaves, and the plants were incubated at 22–24°C under a 16 h light/8 h dark photoperiod for 2–3 days. The coding sequences of the testers were cloned into the pCR8/GW/TOPO vector (Invitrogen) for sequencing. The constructs were further recombined into pEarleyGate201-YN or pEarleyGate202-YC and fused to YFP^N^ or YFP^C^, respectively, and used for BiFC analysis (Kerppola, 2006). The reconstituted YFP signals were observed using a confocal microscope (TCS SP5; Leica).

### 2.9 Pull-down assay and western blotting

Pull-down experiments were performed as previously described (Chen et al., 2018). GST-fused peptides for GLR3.6 and 6xHis-14-3-3ω (50 μg each) were mixed with Ni-NTA agarose and incubated at 4°C for 2 h. The His-tagged pulled-down proteins were collected and subjected to 10% SDS-PAGE. Western blotting was performed as previously described (Chen et al., 2018). Anti-GFP antibody (Abcam; ab290; 1:10000), Anti-GST antibody (Proteintech; 66001-1-lg; 1:2000), and anti-6xHis antibody (GeneTex; GTX628914; 1:3000) were used for immunoblotting. The signals were detected through enhanced chemiluminescence using the CDP-Star reagent (Amersham Biosciences).

### 2.10 Statistical analyses

Each experiment was repeated at least thrice. Values are expressed as the mean ± SD of the gene expression and phenotypic analyses. Statistical analysis was performed using the Student’s *t* test and one-way analysis of variance with *post-hoc* Tukey’s HSD test. Statistical significance was set at *P* < 0.05.

### 2.11 Primers and accession numbers

Primers for cloning, RT-PCR, and q-PCR, as well as accession numbers of genes, are in **Supplementary Table S1**.

## 3 Results

### 3.1 Similarity in the protein structures of GLR3.6 and GLR3.7

**Figure 1:**
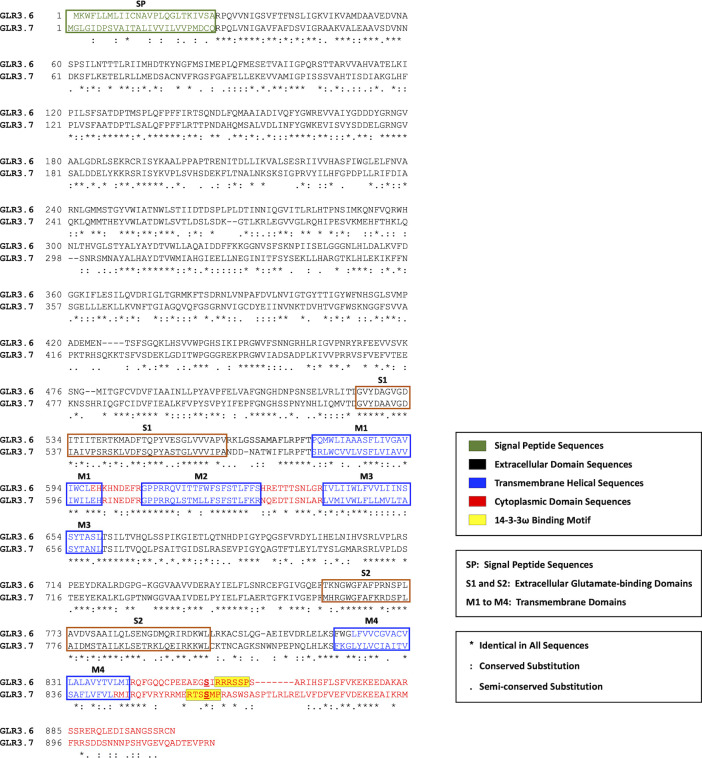
Sequences alignment of GLR3.6 and GLR3.7. The plasma membrane localization signal peptide (SP; green coded), extracellular domain (black coded), transmembrane helix (purple coded), and cytoplasmic domain (red coded) are shown. The extracellular glutamate binding domains (S1 and S2) and four transmembrane domains (M1 to M4) are indicated; M2, an ion-pore loop does not span the membrane. The sequences and protein structures of GLR3.6 and GLR3.7 are similar (45.2% identity). The 14-3-3ω-binding motif is highlighted in yellow shading. Consensus symbols of (*) identical, (:) conserved, and (.) semi-conserved residues are as indicated. Amino acid sequences were aligned with the use of ClustalW (https://www.genome.jp/tools-bin/clustalw).

Sequence alignment and motif structure analysis reveals a similarity in the protein sequences and secondary structure of GLR3.6 and functional GLR3.7 (**Figure 1**). Functionally, GLR3.7 is involved in response to salt stress by modulating Ca^2+^ signaling (Wang et al., 2019). Similar to the structure of animal iGluRs, Arabidopsis GLR3.6 and GLR3.7 harbor a potential plasma membrane-localized signal peptide (SP), three transmembrane domains (M1, M3, and M4), and an ion-pore loop (M2). These subunits combine into a functional tetramer. The putative ligand-binding domain is formed by interactions between the S1 and S2 domains (Colquhoun and Sivilotti, 2004).

A previous study by Wudick et al. (2018) hypothesized based on the analysis of Mode 1 (K/RXXS^p^/T^p^XP) or Mode 2 (K/RXXXS^p^/T^p^XP) in the C-terminal part of GLR3.6, which confirmed a canonical 14-3-3ω binding motif (amino acids 858-RRRSSP-863; **yellow shading**), and our result showed that the Ser860 of GLR3.7 were not aligned with the predicted 14-3-3ω binding motif of GLR3.6. Phosphorylation of Ser860 of GLR3.7 by CDPK16 is required for its functions. In addition, Ser860 of GLR3.7 is also phosphorylated by CDPK3 and CDPK34 (Wang et al., 2019).

### 3.2 CDPK16 specifically phosphorylates Ser856 of GLR3.6 *in vitro*

**Figure 2:**
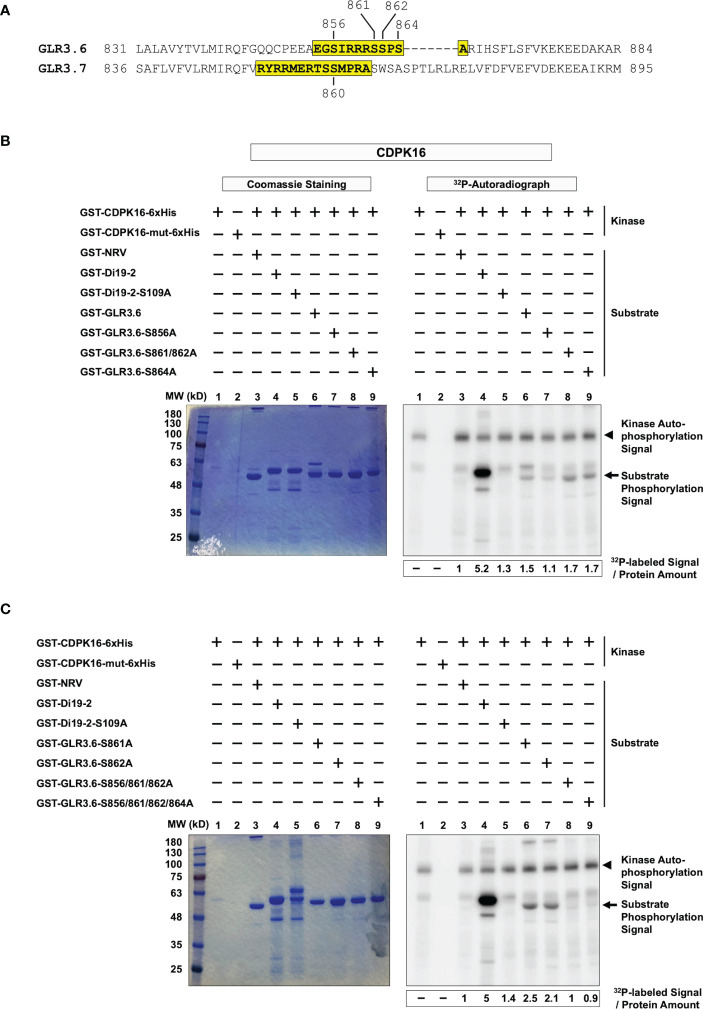
CDPK16 phosphorylates S856 of GLR3.6 *in vitro*. **(A)** The yellow-shaded fragments of GLR3.6 and GLR3.7 containing the 14-3-3ω binding motif were fused to GST then used as substrates for CDPK16 phosphorylation kinase assay. The Ser860 residue of GLR3.7 is phosphorylated by CDPK16, CDPK3, and CDPK34. The Ser856, Ser861, Ser862, and Ser864 residues of GLR3.6 are highlighted. They were tested for phosphorylation by CDPK16. Recombinant kinase-active GST-CDPK16-6xHis and kinase-dead GST-CDPK16-mut-6xHis were used to perform the kinase assay *in vitro*. **(B)** and **(C)** Substrates of the GST-fused peptides (5–10 μg) of GLR3.6, GLR3.6-S856A, GLR3.6-S861/862A, GLR3.6-S864A, GLR3.6-S861A, GLR3.6-S862A, GLR3.6-S856/861/862A, and GLR3.6-S856/861/862/864A, were analyzed. The well-known substrates GST-Di19-2 and GST-Di19-2-S109A were used as a positive and negative control, respectively. GST-NRV was used as a glutathione S-transferase (GST) tag control. Coomassie blue-staining gel shows the protein input (Left panels), and the corresponding ^32^P-labeled autoradiograms (Right panels) are shown. The autophosphorylation signal of CDPK16 and the substrate phosphorylation signals are marked with arrowheads and arrows, respectively. The specific ^32^P-labeling intensity was calculated based on γ-^32^P-labeled signals normalized to the band intensity of input with CBB staining.

We tested whether CDPK16 phosphorylates Ser856, -861, -862, or -864 residues of GLR3.6 *in vitro* (**Figure 2A**). Peptide fragments of GLR3.6 (amino acids 854-EGSIRRRSSPSA-865) and GLR3.7 (amino acids 851-RYRRMERTSSMPRA-864; used for reference) (**Figure 2A**, yellow shaded) and their Ser to Ala (i.e., S to A)-mutated variant (**Supplementary Table S2**) were fused with glutathione S-transferases (GST) and used as a substrate for *in vitro* kinase assay.

The recombinant kinase-active CDPK16 (GST-CDPK16-6xHis) and kinase-dead CDPK16 (CDPK16-S274A) mutant (GST-CDPK16-mut-6xHis; used to clarify the autophosphorylation signals of CDPK16) were subjected to double-affinity protein purification using tags for GST and 6xHis. GST-NRV was used as a GST tag control (Curran et al., 2011; Wang et al., 2019). The GST-fused peptides of the drought-induced 19-2 (GST-Di19-2) and GST-Di19-2-S109A mutant (**Supplementary Table S2**) were used as positive and negative phosphorylation substrates (Curran et al., 2011), respectively, to confirm CDPK16 kinase activity. The results collected from the positive and negative references in the *in vitro* kinase assay (**Figures 2B, C**, lanes 1–5) were consistent with previous reports by Curran et al. (2011) and Wang et al. (2019).

Our results showed that the CDPK16-dependent ^32^P-labeled signal was detected in the peptide fragment of normal GLR3.6 (**Figure 2B**, lane 6), whereas the GLR3.6-S856A mutant largely impaired the phosphorylation signal (**Figure 2B**, lane 7). Notably, the mutants GLR3.6-S861/862A and GLR3.6-S864A retained the ^32^P-labeling (**Figure 2B**, lanes 8–9).

We further analyzed the phosphorylation signals of the single, triple, and quadruple mutations of GLR3.6-S861A, GLR3.6-S862A, GLR3.6-S856/861/862A, and GLR3.6-S856/861/862/864A (**Figure 2C**). The results clearly showed that the ^32^P-labeled signals were impaired specifically by the S856A mutation (**Figure 2C**, lanes 6–9). Thus, the *in vitro* kinase assay confirmed that CDPK16-mediated phosphorylation of GLR3.6 occurred on the Ser856 residue.

### 3.3 Ser856 residue of GLR3.6 is phosphorylated by CDPK16 but not by CDPK3 or CDPK34

**Figure 3:**
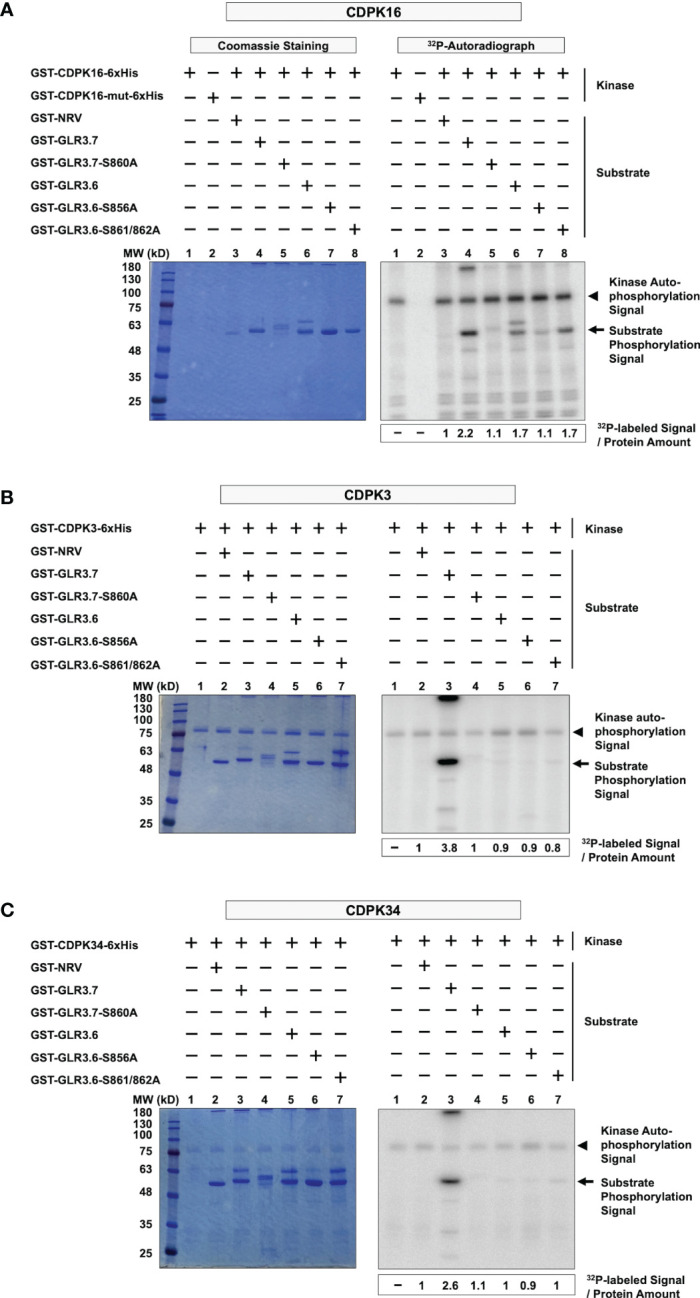
CDPK16 specifically phosphorylates Ser856 of GLR3.6, but CDPK3 and CDPK34 did not. **(A–C)** Recombinant kinases GST-CDPK16-6xHis, GST-CDPK3-6xHis, and GST-CDPK34-6xHis were used to perform the kinase assays *in vitro*. Substrates of the GST-fused peptides GST-GLR3.6, GST-GLR3.6-S856A, and GST-GLR3.6-S861/862A were analyzed. GST-GLR3.7 and GST-GLR3.7-S860A were used as positive and negative control, respectively. GST-NRV was used as a GST tag control. Coomassie blue-staining gels show the protein input (Left panels), and the corresponding ^32^P-labeled autoradiograms (Right panels) are shown. The autophosphorylation signals of CDPK16, CDPK3, and CDPK34 (approximately 100 kD, 85 kD, and 85 kD, respectively), are visualized and are marked with arrowheads. The substrate phosphorylation signals are marked with arrows. The specific ^32^P-labeling intensity was calculated based on γ-^32^P-labeled signals normalized to the band intensity of input with CBB staining.

The Ser860 residue of GLR3.7 is phosphorylated by CDPK16, CDPK3, and CDPK34 (Wang et al., 2019), consistent with the results obtained for CDPK16 (**Figure 3A**, lanes 1–5) and CDPK3 and CDPK34 (**Figures 3B, C**, lanes 1–4); all three kinases phosphorylate the Ser860 residue of GLR3.7. However, we noticed that the autophosphorylation signal pertaining to CDPK16 was stronger than that of CDPK3 and CDPK34.

Interestingly, our results revealed kinase-specific differences in the phosphorylation of Ser856 of GLR3.6. Ser856 of GLR3.6 was phosphorylated by CDPK16 (**Figure 3A**, lanes 6–8), but neither CDPK3 nor CDPK34 did so (**Figures 3B, C**, lanes 5–7). This result implies that CDPK16 exclusively phosphorylates Ser856 in GLR3.6.

### 3.4 Protein-protein interaction between GLR3.6 and 14-3-3ω

**Figure 4:**
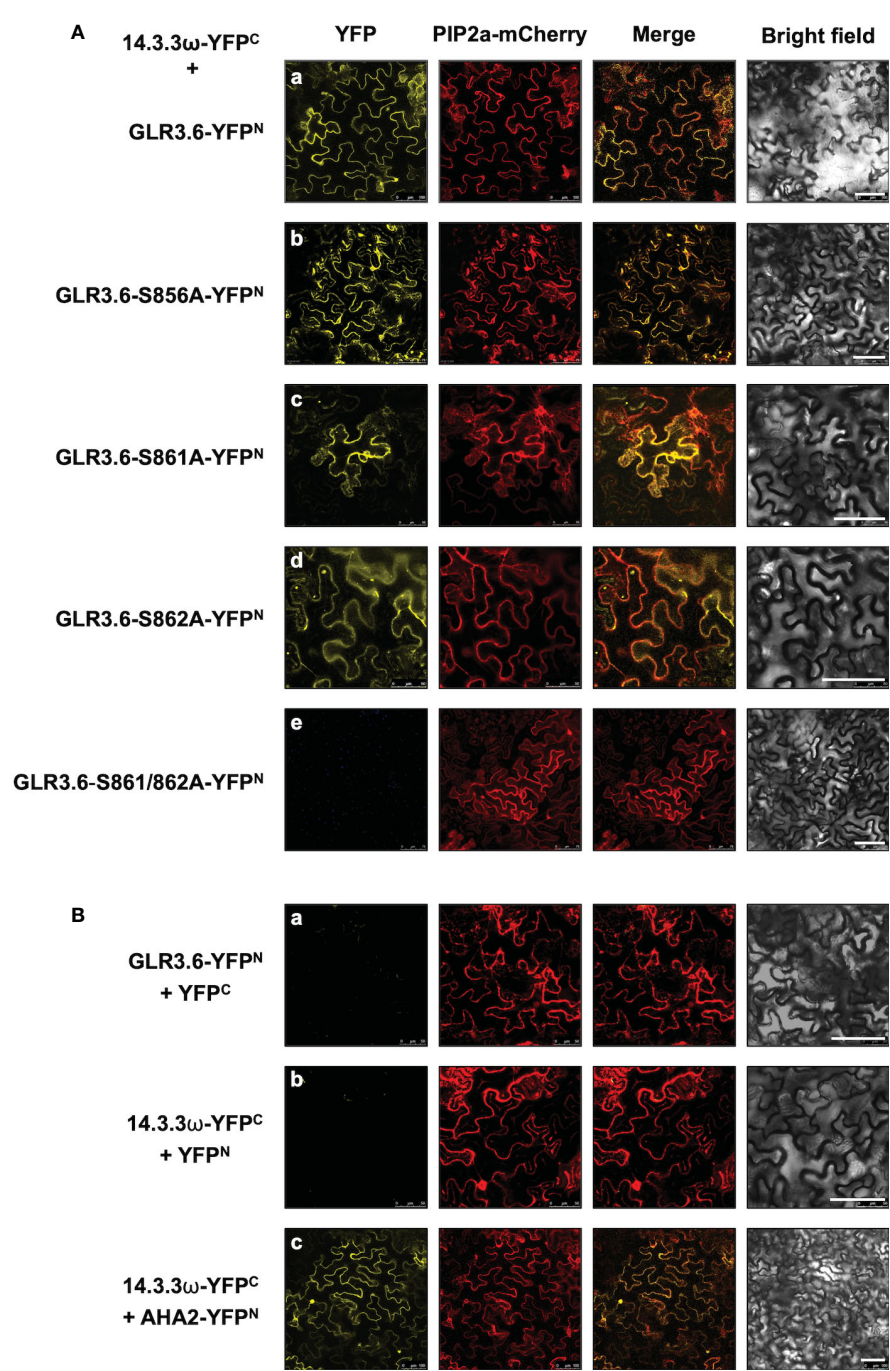
GLR3.6 interaction with 14-3-3ω in the plasma membrane was analyzed in tobacco cells. The bimolecular fluorescence complementation (BiFC) assay was used to analyze the protein–protein interactions. Confocal microscopy was used to detect the reconstitute yellow fluorescence protein (YFP) signal. **(A)** The 14-3-3ω-YFPC was co-expressed with (a) GLR3.6-YFP^N^, (b) GLR3.6-S856A-YFP^N^, (c) GLR3.6-S861A-YFP^N^, (d) GLR3.6-S862A-YFP^N^, or (e) GLR3.6-S861/862A-YFP^N^ in tobacco cells. **(B)** The interactions of (a) GLR3.6-YFP^N^ with YFP^C^ and (b) YFP^N^ with 14-3-3ω-YFP^C^ were used as negative controls. (c) The well-known AHA2-YFP^N^ interaction with 14-3-3ω-YFP^C^ was used as positive control The PIP2a-mCherry was used as the plasma membrane marker. Scale bars = 100μm.

Phosphorylation of the Ser860 residue of GLR3.7 is required for the interaction with 14-3-3ω (Wang et al., 2019). A bimolecular fluorescence complementation (BiFC) assay was performed to confirm the interaction between 14-3-3ω and the plasma membrane-localized GLR3.6. The Ser-to-Ala mutations of GLR3.6 were also analyzed in tobacco (*Nicotiana benthamiana*) cells (**Figure 4**).

The reconstituted yellow fluorescent protein (YFP) signals were observed in the plasma membrane following the interaction of 14-3-3ω with GLR3.6 and mutant GLR3.6-S856A (**Figure 4A, panels a and b**). The single mutants GLR3.6-S861A and GLR3.6-S862A interacted with 14-3-3ω in a manner similar to that of GLR3.6 (**Figure 4A, panels c and d**). Notably, the GLR3.6-S861/862A double mutant did not interact with 14-3-3ω (**Figure 4A, panel e**). PIP2A-mCherry was used as the plasma membrane marker. The transiently expressed proteins were detected by immunoblotting using anti-GFP antibodies (**Supplementary Figure S2**)

The interactions between GLR3.6-YFP^N^ and YFP^C^ and 14-3-3ω-YFP^C^ and YFP^N^ were used as negative controls (**Figure 4B, panels a and b**). The well-known plasma membrane H^+^-ATPase AHA2 was used as a positive control for analyzing the 14-3-3ω interaction (**Figure 4B, panel c**), which was reported in the plasma membrane (Wang et al., 2019).

**Figure 5:**
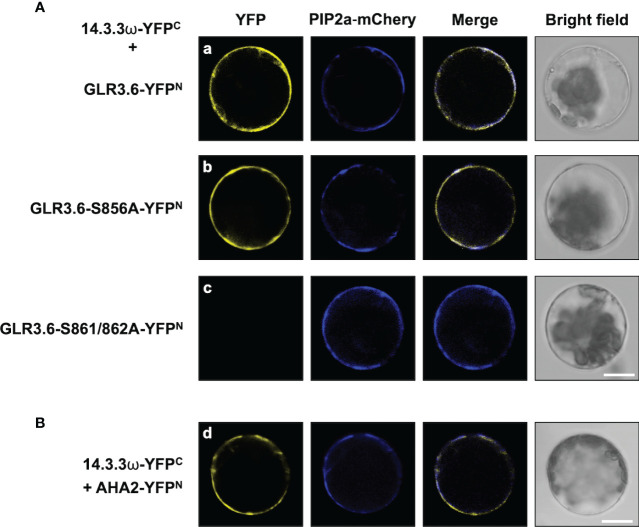
GLR3.6 interaction with 14-3-3ω in the plasma membrane was analyzed in Arabidopsis cells. BiFC assay was used to analyze the protein–protein interaction. Confocal microscopy was used to detect the reconstitute YFP fluorescence signal. **(A)** 14-3-3ω-YFP^C^ was co-expressed with (a) GLR3.6-YFP^N^, (b) GLR3.6-S856A-YFP^N^, or (c) GLR3.6-S861/862A-YFP^N^ in Arabidopsis protoplast cells. **(B)** The interaction between (d) AHA2-YFP^N^ with 14-3-3ω-YFP^C^ was used as positive control. Scale bars = 20 μm.

**Figure 6:**
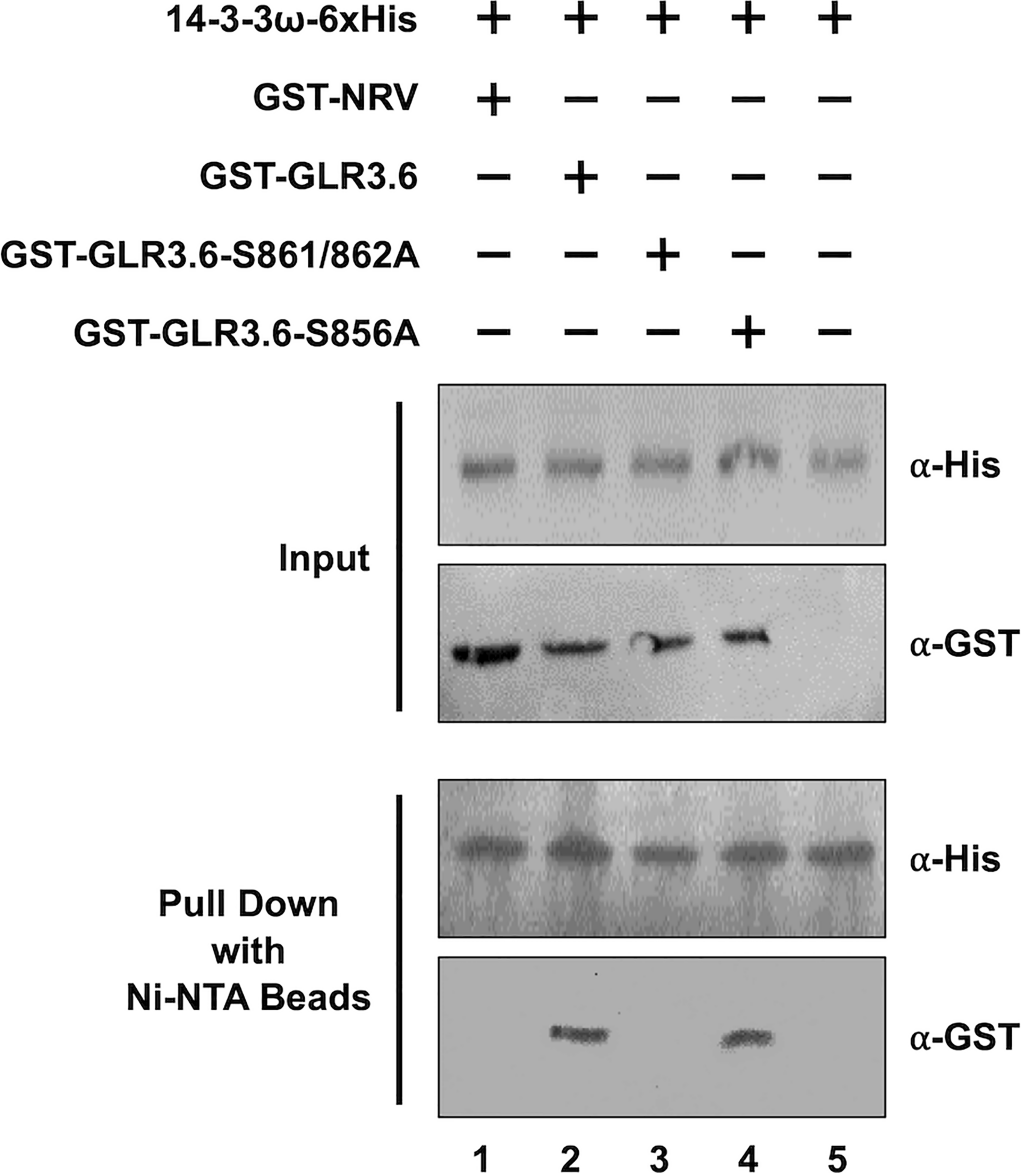
GLR3.6 interaction with 14-3-3ω was verified *in vitro* using pull-down assay. Recombinant 6xHis-14-3-3ω mixed with GST-NRV and GST-fused peptide fragments of GST-GLR3.6, GST-GLR3.6-S861/862A, or GST-GLR3.6-S856A were analyzed by western blotting using the antibodies α-His and α-GST (Input). GST-NRV was used as GST tag control. 6xHis-14-3-3ω and testers were incubated with the Ni-NTA beads then the pull-down proteins were subjected to western blotting using the antibodies α-His and α-GST (Pull Down).

In addition, the interaction between 14-3-3ω and GLR3.6 and its mutants were analyzed using Arabidopsis protoplast cells (**Figure 5**) and by performing an *in vitro* pull-down assay (**Figure 6**). The results of the BiFC analysis in Arabidopsis protoplast cells were consistent with those observed in tobacco cells. 14-3-3ω interacted with GLR3.6 and GLR3.6-S856A single mutant (**Figure 5A, panels a and b**), but not with the GLR3.6-S861/862A double mutant (**Figure 5A, panel c**).

For the pull-down assay, GST-NRV and GST-fused peptide fragments of GST-GLR3.6, GST-GLR3.6-S861/862A, and GST-GLR3.6-S856A were mixed with 6xHis-14-3-3ω protein (**Figure 6**, Input). GST-NRV was used as the GST-tag control. The His-pull-down results confirmed that 14-3-3ω could interact with GLR3.6 and GLR3.6-S856A single mutant (**Figure 6**, Pull down; lanes 2 and 4) but not with the GLR3.6-S861/862A double mutant (**Figure 6**, Pull down; lane 3), which correlated with the results of the BiFC analysis in tobacco and Arabidopsis cells.

Thus, the results indicate that the unknown kinase phosphorylated Ser861/862 residues of GLR3.6 is essential for interaction with 14-3-3ω.

### 3.5 Negative regulation of GLR3.6 in response to salt stress

**Figure 7:**
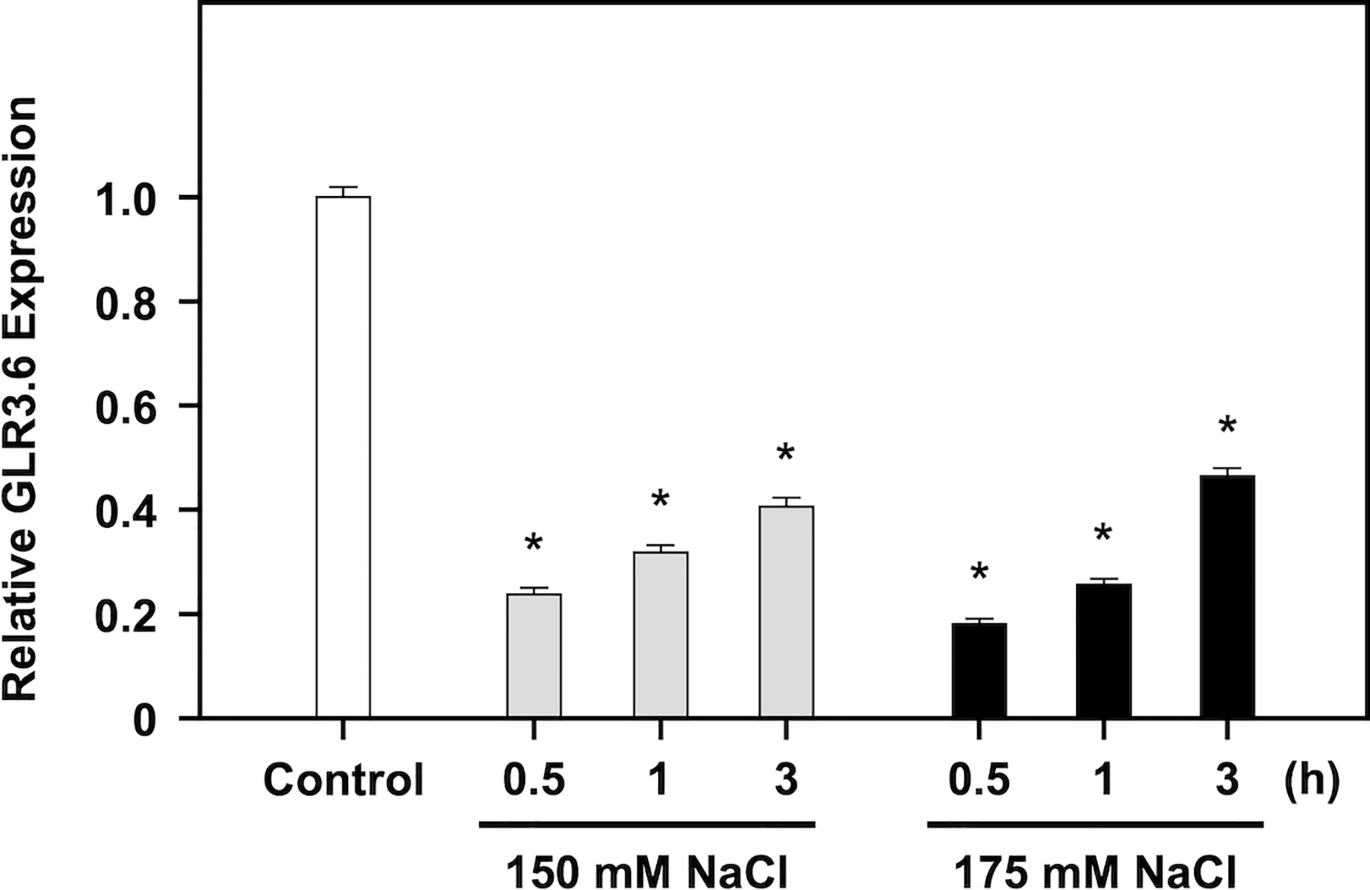
Negative regulation of *GLR3.6* expressions on salt treatment. Seven-day-old seedlings were treated without or with 150 mM and 175 mM NaCl for 0.5, 1, and 3 h, respectively. The transcription level of *GLR3.6* was analyzed using q-PCR. The fold change expression level was normalized relative to that of the control. The data are presented as mean values ± SD. *N* = 3. The asterisks indicate significant differences between samples (*P* < 0.05, student’s *t* test). *ACTIN2* was used as the input control for transcript level normalization.

GLR3.6 plays a positive role in controlling root development, and *glr3.6* mutant showed a phenotype with shorter root length under normal growth conditions (Singh et al., 2016). We showed that the expression levels of GLR3.6 were rapidly and significantly downregulated in response to 150 and 175 mM of NaCl treatment for 30 min (**Figure 7**), when analyzed using quantitative real-time PCR (q-PCR). These results imply that GLR3.6 plays a role in quick response to salt stress.

### 3.6 Overexpression of GLR3.6-S856A mutant shows a less salt-responsive phenotype

**Figure 8:**
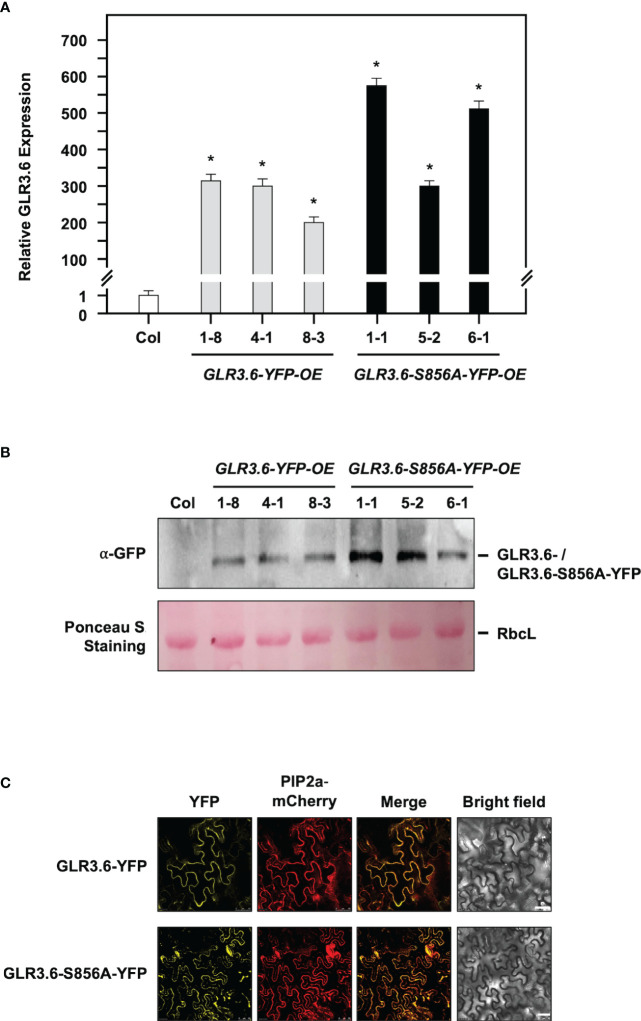
Characterization of YFP-fussed GLR3.6- and GLR3.6-S856A-overexpression lines. **(A, B)** Seven-day-old seedlings were used for GLR3.6 accumulation assay, which was analyzed using q-PCR and western blotting. Antibodies against α-GFP were used for protein accumulation assay. The ribulose bisphosphate carboxylase large subunit stained using Ponceau S was used as loading control. **(C)** Subcellular localization of the YFP-fused GLR3.6 and GLR3.6-S856A was found to be completely localized to the plasma membrane using transient expression assay. Scale bars = 50 μm. The data are presented as mean values ± SD. N = 3. The asterisks (*) indicate significant differences between samples (P < 0.05, student's t test). ACTIN2 was used as the input control for transcript level normalization. μm.

Arabidopsis GLR subunits combine into a functional tetramer similar to iGluRs (Colquhoun and Sivilotti, 2004). We characterized two *glr3.6* mutants (**Supplementary Figure S1**), three YFP-fused *GLR3.6*-overexpression (OE) lines (*GLR3.6-YFP-OE-1-8, OE4-1, OE8-3*) and *GLR3.6-S856A-YFP-OE* lines (*GLR3.6-S856A-YFP-OE1-1, OE5-2, OE6-1*) based on the results of q-PCR analysis (**Figure 8A**) and western blotting assay (**Figure 8B**). Additionally, we confirmed that they were localized to the plasma membrane by performing a transient expression assay (**Figure 8C**).

**Figure 9:**
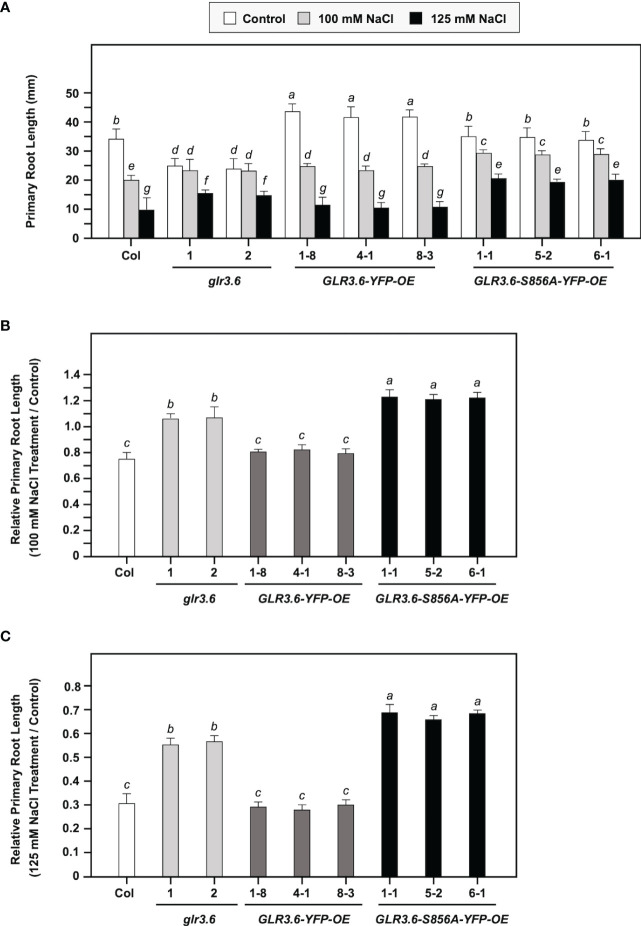
Phenotypes of *glr3.6*, GLR3.6-YFP, and GLR3.6-S856A-YFP-overexpression plants under normal growth conditions and salt stress. Four-day-old seedlings with similar root lengths were transferred to 1/2 MS plates without and with 100 mM NaCl or 125 mM NaCl for 6 days, respectively. **(A)** The primary root length was measured. **(B, C)** Relative primary root length (NaCl treatment/control) was calculated. The data are presented as mean values ± SD. *N* = 3. Various letters indicate significant differences compared with Col plants and control (*P* < 0.05, Turkey’s-test one-way ANOVA).

In this study, four-day-old seedlings with similar root lengths were transferred to 1/2 MS plates without (Control) or with 100 or 125 mM NaCl for six days. Under normal growth conditions (**Figure 9A**, Control), *glr3.6* mutants showed shorter primary root lengths, *GLR3.6-YFP-OE* plants showed longer primary root lengths; however, *GLR3.6-S856A-YFP-OE* plants showed no significant differences compared with that of the Col plants (**Supplementary Figure S3**). These results are consistent with previous reports that GLR3.6 is a positive factor required for controlling root length (Singh et al., 2016).

We calculated relative primary root growth in response to 100 mM or 125 mM NaCl stress (NaCl treatment/control) for six days (**Figures 9B, C**). The *glr3.6* mutants and *GLR3.6-S856A-YFP-OE* plants showed less sensitivity to salt stress, and *GLR3.6-YFP-OE* plants showed no significant difference compared with the Col plants. This result indicates that the overexpression of *GLR3.6-S856A* might have a dominant negative effect that presented a less salt-responsive phenotype.

In conclusion, GLR3.6 is essential for the control of root growth under normal growth conditions, and Ser856 of GLR3.6 plays a role in response to salt stress.

## 4 Discussion

The 14-3-3 scaffold proteins interact with phosphorylated serine or threonine residues in specific motifs of client proteins, leading to changes in the client protein such as in structure, activity, localization, and association with larger protein complexes (Johnson et al., 2002; Fuglsang et al., 2003; Paul et al., 2005; Mayfield et al., 2012). Previous studies have demonstrated that interactions with 14-3-3 play a role in the activities of shaker-type K^+^ (GORK) and Ca^2+^ channels, which control K^+^ and Ca^2+^ influx during processes related to ABA and salt response, seed germination, and primary root growth control (Mayfield et al., 2012; van Kleeff et al., 2018; Chang et al., 2019; Yang et al., 2019; Chen et al., 2021; Huang et al., 2022). Ca^2+^-conducting factor GLR3.6 controls root development (Singh et al., 2016). In this study, we showed that GLR3.6 shares similar protein structures to the known functional GLR3.7, which is involved in seed germination, pollen tube growth, salt stress, and ABA signaling (**Figure 1**), implying that GLR3.6 is a plasma membrane-localized Ca^2+^-permeable channel that affects Ca^2+^ signaling (**Figures 4** and **5**). We demonstrated that GLR3.6 plays a positive role in root length control both under normal growth conditions and in response to salt stress (**Figure 9A**).

Ca^2+^ is a ubiquitous signaling molecule and acts as a second messenger in eukaryotic cells that participate in nearly all aspects of plant growth and development (Park and Shin, 2022). When signaling takes place, Ca^2+^ either enters the cell through the ion channels on the plasma membrane or is triggered to release from its stored form (Luan and Wang, 2021). In plants, Ca^2+^-binding proteins function as a Ca^2+^ sensor that detects changes in Ca^2+^ levels by binding with domains like EF-hands. Our previous study revealed that Ser860 of GLR3.7 is phosphorylated by CDPK3, CDPK16, and CDPK34 (Wang et al., 2019). In the present study, Ser856 in GLR3.6 was discovered to be phosphorylated by CDPK16 and not by CDPK3 and CDPK34 (**Figures 2** and **3**). CDPK16 is the most distinct protein kinase among CDPKs in Arabidopsis (Harper et al., 2004; Curran et al., 2011). A previous study showed that among 103 substrates by representative CDPKs of CDPK1 (subgroup-I), CDPK34 (subgroup-II), and CDPK10 (subgroup-III) found that 93 of the phosphorylated substrates were not recognized by CDPK16 (subgroup-IV). This suggests substrate specificity in CDPK16 (Curran et al., 2011). The results also showed that CDPK16 phosphorylated Ser109 of Di19-2, which was not recognized by CDPK1, CDPK10, or CDPK34 (Curran et al., 2011).

In Arabidopsis, a number of common CDPK phosphorylation motifs have been identified (Harper et al., 1994). The first consensus phosphorylation motif is ϕ_-5_-X_-4_-Basic_-3_-X_-2_-X_-1_-*S*, where X can be any residue and ϕ is a hydrophobic residue. The underlined *S* is phosphorylated in this motif. Basic_-9_-Basic_-8_-X_-7_-Basic_-6_-ϕ_-5_-X_-4_-X_-3_-X_-2_-X_-1_-*S*-X_+1_-Basic_+2_ is the second consensus phosphorylation motif. The ϕ_-3_-R_-2_-ϕ_-1_-*S*-ϕ_+1_-X_+2_-K_+3_-R_+4_ is the third consensus phosphorylation motif, and the *S*-X_+1_-R_+2_ is the fourth consensus phosphorylation motif (Curran et al., 2011; Huang et al., 2013; Wang et al., 2019). Arabidopsis GLR3.6 Ser856 and Ser862 perfectly match the fourth (856-*S* IR-858) and first (857-IRRRS *S*-862) consensus phosphorylation pattern, respectively. CDPK16 phosphorylated Ser856 of GLR3.6, and thus, has been revealed to be a novel specific substrate for CDPK16 *in vitro*. However, it is critical to determine whether Ser856 of GLR3.6 is a CDPK16 substrate *in vivo*. We tried the in-planta phosphorylation assay using the phosphor-Ser/Thr antibody as previously described (Hu et al., 2021), but still, we were unable to detect the signal. It is may due to technical limitations. Further research is necessary.

14-3-3 binds to its partners in a phosphorylation-dependent and independently (Fuglsang et al., 2007). Despite the fact that 14-3-3 binds to the phosphopeptide ligand and residues in the groove of 14-3-3 also leads to additional interactions with this specific site and the target proteins. Furthermore, 14-3-3 binds to several sites on a single protein that contains single phosphorylated Ser/Thr residues (Alsterfjord et al., 2004; Fuglsang et al., 2007; Rong et al., 2007). We analyzed the C-terminal end of GLR3.6, harboring the 858-RRRSSP-863 sequence (**Figure 1**), which fits the consensus binding motif of Mode 1 or 2 of 14-3-3ω (Wudick et al., 2018). In this study, we showed that Ser861 and Ser862 residues of GLR3.6 are required for interacting with 14-3-3ω, and the GLR3.6-S861/862A double mutant does not partake in this interaction (**Figures 4** **–** **6**). In fact, the phosphorylation of amino acid residues inside or outside the 14-3-3-binding motif by CDPKs and other protein kinases has a significant influence on the interaction between 14-3-3 and client proteins (Yaffe et al., 1997; Johnson et al., 2002; Shen et al., 2003; Shin et al., 2011; van Kleeff et al., 2018).

Arabidopsis GLR3.6-Ser862 perfectly matches the first consensus phosphorylation motif of CDPKs. In this study, we showed that Ser861 and Ser862 of GLR3.6 were not recognized by CDPK1 (data not shown), CDPK3, CDPK16, and CDPK34 (**Figures 2** and **3**). Additionally, CDPK16-induced phosphorylation of Ser856 in GLR3.6, which is outside the 14-3-3ω binding motif, the GLR3.6-S856A single mutation does not affect the 14-3-3ω-related interaction (**Figures 4** **–** **6**). These suggested that they are phosphorylated by uncharacterized CDPKs or other protein kinases for fostering interaction with 14-3-3ω. However, to understand more about the mechanism underlying their interaction and their impact on Ca^2+^ channel activity, the transgenic *GLR3.6-S861/862A* mutation plants are needed for further studies.

Clade III GLR proteins are involved in Arabidopsis growth and development. The expression of *GLR3.7* is positively regulated by salt stress (Cheng et al., 2016), but *glr3.7* and *GLR3.7-OE* plants do not show a significant difference in root growth control under normal growth conditions and in response to salt stress, as compared to WT (Wang et al., 2019). And, the phosphorylation of Ser860 residue of GLR3.7 by CDPKs is an important step in ABA signaling and salt stress response (Wang et al., 2019; Chen et al., 2021). Previous research found that *GLR3.6*-mutated plants largely impaired cytosolic Ca^2+^ levels and influenced primary root length and lateral root density (Singh et al., 2016). In this study, we showed that the expression level of *GLR3.6* was rapidly suppressed in response to salt stress (**Figure 7**). The *GLR3.6-S856A-OE* plants displayed a primary root growth phenotype less sensitive to salt stress, similar to *glr3.6*. However, the *GLR3.6-S856A-OE* plants did not exhibit any significant defects under normal growth conditions (**Figure 9**). It suggests that GLR3.6 mediates [Ca^2+^]_cyt_ levels and impacts root growth in Arabidopsis under normal growth conditions. Besides, *glr3.6* mutants and *GLR3.6-S856A-OE* plants exhibit salt-less sensitive root growth phenotype, implying that salt-mediated [Ca^2+^]_cyt_ levels influence root growth in the *glr3.6* mutants and *GLR3.6-S856A-OE*. However, [Ca^2+^]_cyt_ levels are required to comprehend the function of GLR3.6; thus, would be interesting to generate the transgenic plants of *glr3.6* mutants harboring aequorin (a Ca^2+^-sensitive photoprotein) for future studies.

It has been known that GLR3.6 can interact with GLR3.6 itself and with GLR3.4 by yeast-2-hybrid assay (Price et al., 2013). In our BiFC analysis, we also found that GLR3.6 can interact with itself and with GLR3.7 (**Supplementary Figure S4**). The result implies the homo or hetero-multimeric of GLR3.6 with other GLRs, e.g., GLR3.4 and GLR3.7, in the checking and balancing of functioning channel activity in response to salt-inducing signals.

## 5 Conclusion

**Figure 10:**
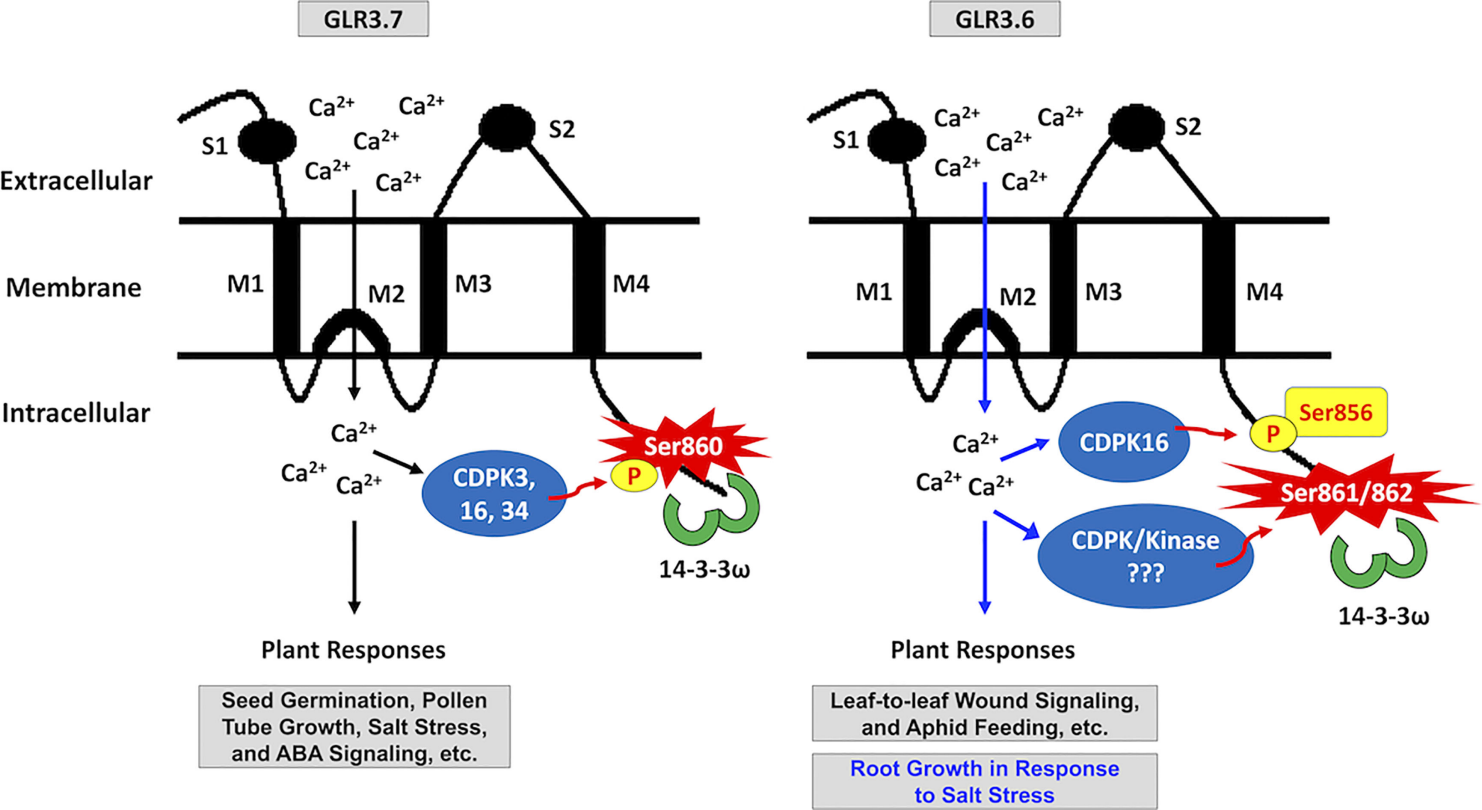
Schematic model of the 14-3-3ω binding site in clade-III GLR family members GLR3.6 and GLR3.7. S860 of GLR3.7 is phosphorylated by CDPK3, CDPK16, and CDPK34, which bind with 14-3-3ω to regulate ABA and salt response, seed germination, and root growth. This study postulated that S861/862 residues of GLR3.6 may be phosphorylated by uncharacterized CDPK and protein kinase, enabling the binding with the 14-3-3ω. Notably, the CDPK16 specifically phosphorylates Ser856 residue of GLR3.6, but CDPK3 and CDPK34 do not. Additionally, Ser856 participates in salt stress-responsive primary root growth.

We showed through *in vitro* experiments that the Ser856 residue of GLR3.6 is specifically phosphorylated by CDPK16 kinase but not by CDPK3 and CDPK34. The GLR3.6-S861/862A mutations in the 14-3-3 binding site abolished the interaction with 14-3-3ω, whereas the GLR3.6-S856A mutation had no effect on the receptor’s interaction with 14-3-3ω (**Figure 10**). Here, we confirmed that GLR3.6 is a positive factor required for root length control under normal growth conditions. The *glr3.6* mutants and *GLR3.6-S856A-OE* lines displayed a less sensitive phenotype in root growth in response to salt stress, confirming the important role played by the Ser856 residue; however, the effects of 14-3-3ω interaction on the functional control of GLR3.6 and the Ca^2+^ channel activity of these sites remain to be uncovered. In conclusion, the phosphorylation of Ser856 by CDPK16 in GLR3.6 is a factor in root-growth control under normal growth conditions as well as in response to salt stress.

## 6 Author’s note

This paper is dedicated to the memory of the late Dr. Ing-Feng Chang

## Data availability statement

The original contributions presented in the study are included in the article/**Supplementary Material**. Further inquiries can be directed to the corresponding author.

## Author contributions

DS, I-F C, and T-L J designed research, DS performed research, DS, and T-L J analyzed data and wrote the article. All authors contributed to the article and approved the submitted version.
