# Supplementary material for: Calcium-dependent protein kinase CDPK16 phosphorylates serine-856 of glutamate receptor-like GLR3.6 protein leading to salt-responsive root growth in Arabidopsis#
Source: Front Plant Sci. 2023 Feb 3;14:1093472. doi: 10.3389/fpls.2023.1093472 (PMC9935832; doi:10.3389/fpls.2023.1093472)
Supplement: Supplementary file 1 [file DataSheet_1.pdf]

Supplementary Table S1. Primers for genotyping, cloning, RT-PCR, and q-PCR, as well as accession numbers of genes. Gray shadings represent restriction enzymes Asc1 and Not1 cutting sites.

Gene (Purpose)	Primer	Sequence (5' to 3')
glr3.6-1 (Genotyping)	LP1	TTCGTTCAAAGGTGGCATAAC
	RP1	CGACTATGAGGAAAGACGCAG
glr3.6-2 (Genotyping)	LP2	ATAGTCGGTGCTGTCATTTGG
	RP2	TCCCCAAAAGCTCTTAAGCTC
GLR3.6 (CDS)	Fw	ATGAAGTGGTTTCTGCTTATGCTCATCATCTG
	Rv	GTTGCAGCGACTTGAACCATTTGCA
GLR3.6 (RT-PCR)	Fw	GTTACAGGTTCGAAGAGGTAGTC
	Rv	CGACTATGAGGAAAGACGCAG
GLR3.6 (q-PCR)	Fw	CGGGACAAATGGTTGCTTAGA
	Rv	CCACTCCACACACCACAAATAATC
GLR3.6 (Point mutation)	S856A-Fw	GAAGCAGAAGGGGCGATCAGGAGAAGAAGCTCACCATCCGCGCGT
	S856A-Rv	GATACGCGCGGATGGTGAGCTTCTTCTCCTGATCGCCCCTTCTGCTTC
	S861/862A-Fw	GAAGCAGAAGGGTCGATCAGGAGAAGAGCCGCACCATCCGCGCGT
	S861/862A-Rv	GATACGCGCGGATGGTGCGGCTCTTCTCCTGATCGACCCTTCTGCTTC
14-3-3ω (CDS)	Fw	ATGGCGTCTGGGCGTGAAGA
	Rv	CTGCTGTTCCTCGGTCGGTT
14-3-3ω (His-tag)	BamH1-Fw	GGATCCATGGCGTCTGGGCGTGAAGA
	EcoR1-Rv	GAATTCCTGCTGTTCCTCGGTCGGTT

Gene (Purpose)	Primer	Sequence (5’ to 3’)
	**854-EGSIRRRSSPSA-865**	
	Asc1-F	CGCGCCAGCGCCCGGGGAAGGGTCGATCAGGAGAAGAAGCTCACCATCCGCGGC
	Not1-R	GGCCGCCGCGGATGGTGAGCTTCTTCTCCTGATCGACCCTTCCCCGGGCGCTGG
	S856A Asc1-F	CGCGCCAGCGCCCGGGGAAGGGGCGATCAGGAGAAGAAGCTCACCATCCGCGGC
	S856A Not1-R	GGCCGCCGCGGATGGTGAGCTTCTTCTCCTGATCGCCCCTTCCCCGGGCGCTGG
	S861A Asc1-F	CGCGCCAGCGCCCGGGGAAGGGTCGATCAGGAGAAGAGCCTCACCATCCGCGGC
**GLR3.6 (Fusion Peptides)**	S861A Not1-R	GGCCGCCGCGGATGGTGAGGCTCTTCTCCTGATCGACCCTTCCCCGGGCGCTGG
	S862A Asc1-F	CGCGCCAGCGCCCGGGGAAGGGTCGATCAGGAGAAGAAGCGCACCATCCGCGGC
	S862A Not1-R	GGCCGCCGCGGATGGTGCGCTTCTTCTCCTGATCGACCCTTCCCCGGGCGCTGG
	S864A Asc1-F	CGCGCCAGCGCCCGGGGAAGGGTCGATCAGGAGAAGAAGCTCACCAGCCGCGGC
	S864A Not1-R	GGCCGCCGCGGCTGGTGAGCTTCTTCTCCTGATCGACCCTTCCCCGGGCGCTGG
	S861/862A Asc1-F	CGCGCCAGCGCCCGGGGAAGGGTCGATCAGGAGAAGAGCCGCACCATCCGCGGC
	S861/862A Not1-R	GGCCGCCGCGGATGGTGCGGCTCTTCTCCTGATCGACCCTTCCCCGGGCGCTGG
	S856/861/862/864A Asc1-F	CGCGCCAGCGCCCGGGGAAGGGGCGATCAGGAGAAGAGCCGCACCAGCCGCGGC
	S856/861/862/864A Not1-R	GGCCGCCGCGGCTGGTGCGGCTCTTCTCCTGATCGCCCCTTCCCCGGGCGCTGG
	**851-RYRRMERTSSMPRA-864**	
	Asc1-F	CGCGCCAGCGCCCGGGCGGTACAGACGGATGGAGAGAACATCCTCGATGCCACGCGCTGC
**GLR3.7 (Fusion Peptides)**	Not1-R	GGCCGCAGCGCGTGGCATCGAGGATGTTCTCTCCATCCGTCTGTACCGCCCGGGCGCTGG
	S860A Asc1-F	CGCGCCAGCGCCCGGGCGGTACAGACGGATGGAGAGAACATCCGCGATGCCACGCGCTGC
	S860A Not1-R	GGCCGCAGCGCGTGGCATCGCGGATGTTCTCTCCATCCGTCTGTACCGCCCGGGCGCTGG

Gene (Purpose)	Primer	Sequence (5’ to 3’)
ACTIN2 (q-PCR)	Fw	GTGTGTGACAAACTCTCTGGGT
	Rv	AGAGGCATCAATTCGATCACTCAG
ACTIN2 (RT-PCR)	Fw	ATGAAGCACAATCCAAGAGAGGTATTCTTA
	Rv	GAGCTTCTCCTTGATGTCTCTTACAATTTC
PCR8/GW/TOPO (Sequencing)	M13-F	GCATGGATGTTTTCCCAGTCACGACG
pEarleyGate-101 (Sequencing)	35S-promotor-F	GGACGATTCAAGGCTTGCTT
GST-NRV-pGEX-4T-1 (Sequencing)	NRV-F	CCGTGATGCAGAAGAAGACC
pGEX-4T-1 (Sequencing)	pGEX-5’-F	GGGCTGGCAAGCCACGTTTGGTG
pRSETA-6xHis (Sequencing)	p202-F	TAATACGACTCACTATAGGG

Sequence data in this article can be found in the TAIR database under the following accession numbers:

14-3-3ω (At1g78300), AHA2 (At4g30190), CDPK3 (At4g23650), CDPK16 (At2g17890), CDPK34 (At5g19360), Di19-2 (At1g02750), GLR3.6 (At3g51480), and GLR3.7 (At2g32400).

Supplementary Table S2. Fusion peptides used in kinase assay *in vitro*. Gray shadings represent point mutations of serine (S) to alanine (A).

**Name**	**Amino Acid Sequences**
Di19-2	DVLKSEQKEMSYREDPY
Di19-2-S109A	DVLKSEQKEMAYREDPY
GLR3.7	RYRRMERTSSMPRA
GLR3.7-S860A	RYRRMERTSAMPRA
GLR3.6	EGSIRRRSSPSA
GLR3.6-S856A	EGAIRRRSSPSA
GLR3.6-S861A	EGSIRRRASPSA
GLR3.6-S862A	EGSIRRRSAPSA
GLR3.6-S864A	EGSIRRRSSPAA
GLR3.6-S861/862A	EGSIRRRAAPSA
GLR3.6-S856/861/862A	EGAIRRRAAPSA
GLR3.6-S856/861/862/864A	EGAIRRRAAPAA

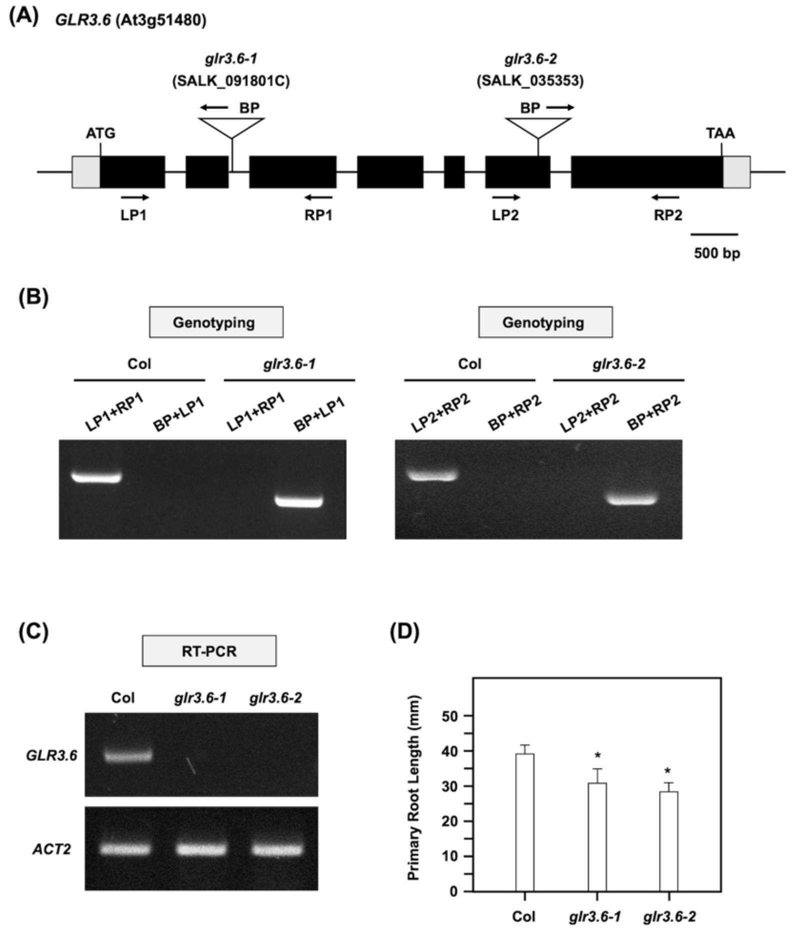

Supplementary Figure S1. Characterization of T-DNA insertion mutants of *glr3.6-1* and *glr3.6-2*. (A) Schematic map of *GLR3.6* gene and T-DNA insertion sites. The grey box, black box, and solid line indicate UTR region, exon, and intron, respectively. T-DNA insertion sites are indicated as triangles. Primers for genotyping are indicated as arrows. ATG and TAA are shown as initiation and stop codons, respectively. **(B)** Genotyping by PCR was analyzed with DNA flanking sequence primers (LP and RP) and T-DNA border primer (BP). **(C)** RT-PCR involved the *GLR3.6* gene-specific primers. *Actin2* (*ACT2*) was used as an internal control. **(D)** Ten-day-old seedlings grown on 1/2 MS plates under normal growth conditions, and then primary root length was measured. The data are presented as mean values ± SD. $N = 3$. *, significant at $P < 0.05$ compared to Col.

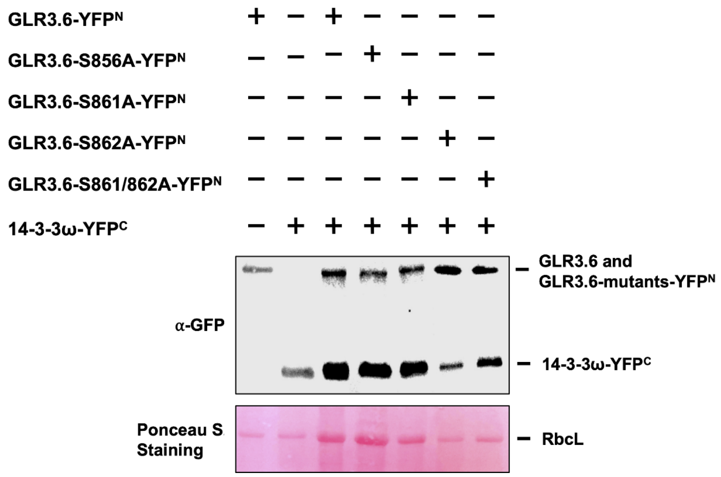

Supplementary Figure S2. Western blotting analysis of protein expression of transgenes 14-3-3ω-YFP^{C}, GLR3.6-YFP^{N}, and GLR3.6-mutants-YFP^{N}. The transgene of GLR3.6-YFP^{N}, 14-3-3ω-YFP^{C}, and 14-3-3ω-YFP^{C} co-expressing with GLR3.6-YFP^{N}, GLR3.6-S856A-YFP^{N}, GLR3.6-S861A-YFP^{N}, GLR3.6-S862A-YFP^{N}, or GLR3.6-S861/862A-YFP^{N}, respectively, in tobacco cells, see **Figure 4**. The transgene expression was analyzed by western blotting using α-GFP antibodies. Mw of 14-3-3ω and GLR3.6 and its mutants are approximately 30 kD and 100 kD, respectively. The ribulose bisphosphate carboxylase large subunit (RbcL) stained with Ponceau S was used as a loading control.

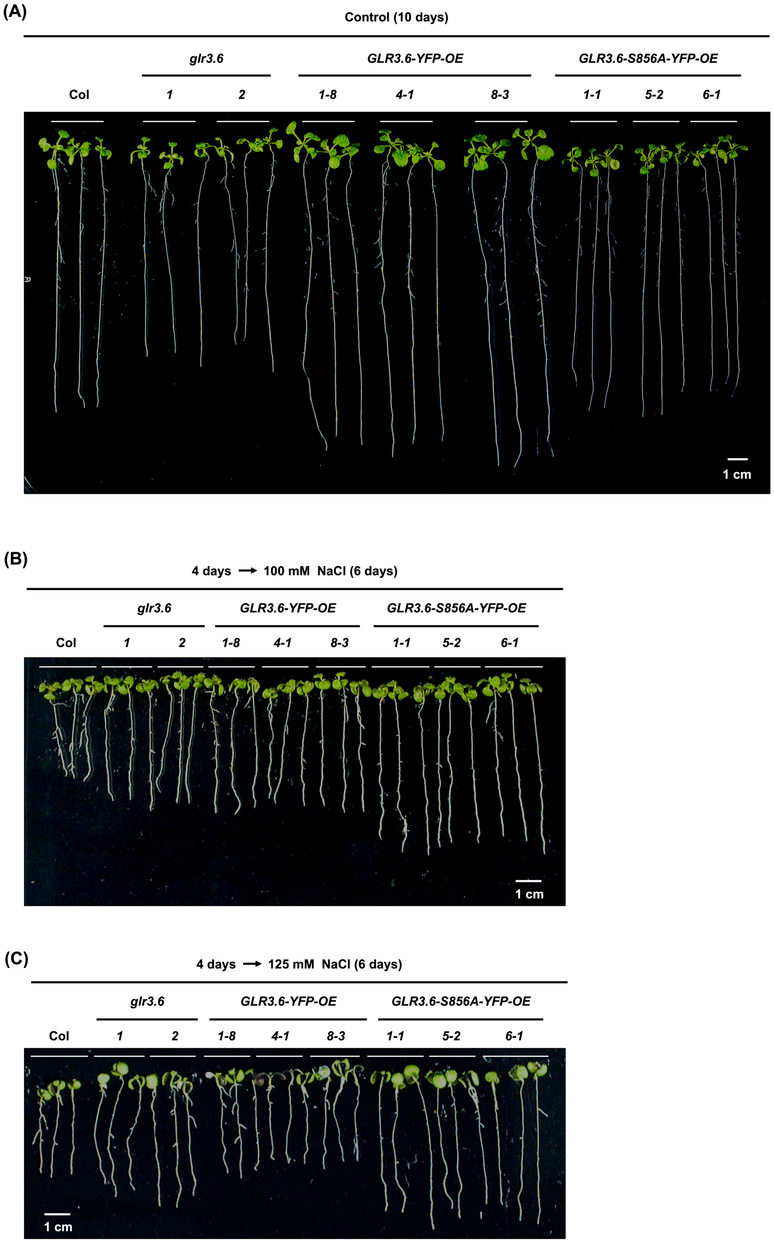

Supplementary Figure S3. Phenotypes of Col, *glr3.6* mutant, *GLR3.6-YFP*-overexpression (OE), and *GLR3.6-S856A-YFP*-OE plants under normal growth conditions and salt stress. (A) to **(C)** Four-day-old seedlings with similar root lengths were transferred to 1/2 MS plates without (control) and with 100 mM or 125 mM NaCl for 6 days, respectively, see **Figure 9**, and were photographed. Bars = 1 cm.

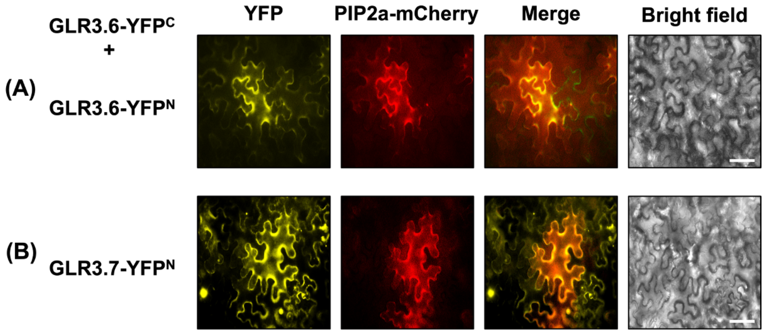

Supplementary Figure S4. GLR3.6 interacts with GLR3.6 and GLR3.7 in the plasma membrane were analyzed in tobacco cells. BiFC assay was used to analyze the protein–protein interaction, as described in **Figure 4**. Fluorescence microscopy was used to detect the reconstitute YFP fluorescence signal. **(A)** and **(B)** The GLR3.6-YFP^{C} co-expressing with GLR3.6-YFP^{N} or GLR3.7-YFP^{N} in tobacco cells, respectively. The PIP2a-mCherry was used as the plasma membrane marker. Bars = 50 μm.

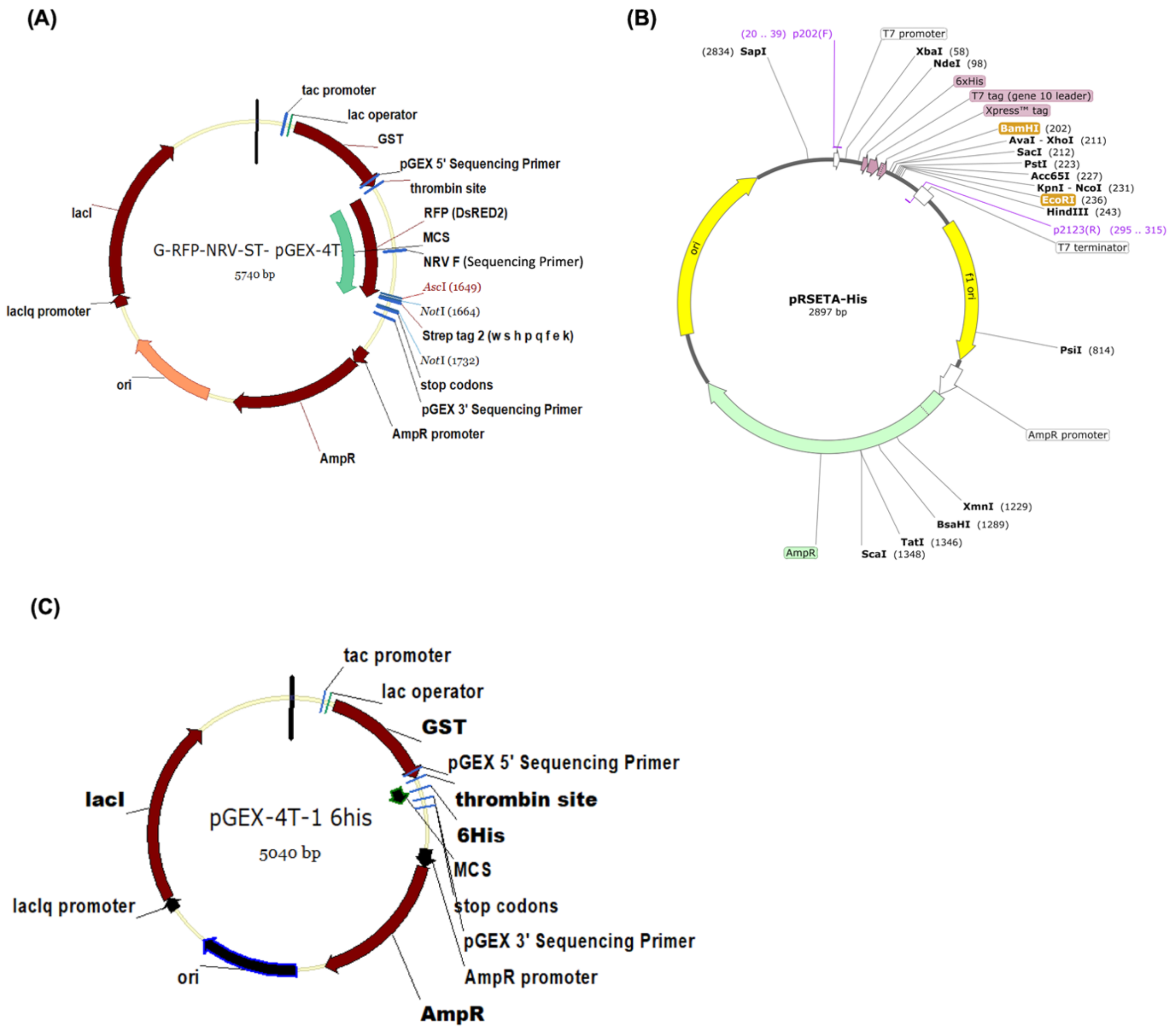

Supplementary Appendix 1. (A) The GST-NRV vector (GST-RFP-NRV-ST-pGEX-4T-1) was modified from the pGEX-4T-1 vector with the insertion of RFP (DsRED2) and Strep-tag II peptide (ST; WSHPQFEK). NRV is a sequencing primer binding site. The GST-NRV vector-only control produces the protein containing GST-RFP-Strep tag II. The self-annealed fusion-peptide primers were designed around 40 to 60 nucleotides, harboring *Asc*I and *Not*I restriction sites for cloning into the GST-NRV vector. **(B)** 14-3-3ω cDNA acquired the *Bam*H1 and *Eco*R1 restriction sites to clone into the pRSETA-6xHis vector. **(C)** The pGEX-4T-1-6xHis vector was modified from the pGEX-4T-1 with an additional 6xHis tag.
